# Improving Nutritive Value of Purple Field Corn Residue and Rice Straw by Culturing with White-Rot Fungi

**DOI:** 10.3390/jof6020069

**Published:** 2020-05-21

**Authors:** Benjamad Khonkhaeng, Anusorn Cherdthong

**Affiliations:** Tropical Feed Resources Research and Development Center (TROFREC), Department of Animal Science, Faculty of Agriculture, Khon Kaen University, Khon Kaen 40002, Thailand; kbenjamad211223@gmail.com

**Keywords:** anthocyanin, *Pleurotus osteratus*, *Volvariella volvacea*, lovastatin, biological treatment

## Abstract

It was hypothesized that white-rot fungus fermented with rice straw and purple field corn improves nutrient utilization via enhanced digestibility and lowers methane (CH_4_) production due to the effects of the lovastatin compound. The aim of the current experiment was to investigate the effect of inoculation of two fungi belonging to white-rot fungus type on feed value and ruminal fermentation characteristic. The experiment was carried out according to a completely randomized 3 × 3 factorial design: three roughage sources (rice straw, purple corn stover, and purple corn field cob) for three inoculation methods (untreated, *P. ostreatus* treated, and *V. volvacea* treated). The two fungi increased concentration of lovastatin when compared to the untreated, and *P. ostreatus* had higher lovastatin production potential than *V. volvacea* (*p* < 0.05). The yield of lovastatin was obtained from rice straw fermentation with *P. ostreatus*. The monomeric anthocyanin content (MAC) in untreated purple field corn cobs was higher than in the fermentation groups. Ruminal fermentation gas production from soluble fractions ranged from −2.47 to 1.14 and differed among the treatments (*p* < 0.01). In comparison to all treatments, the gas production rate for the insoluble fraction was significantly highest (*p* < 0.01) in treatment alone, in which purple field corn stover was fermented with *P. ostreatus* and *V. volvacea*. There was significant interaction in in vitro dry matter digestibility at 12 h of incubation. Purple field corn cob had a higher significant effect on in vitro DM digestibility at 12 and 24 h after incubation when compared to that of other groups. Moreover, current research has found that roughage fermented with *P. ostreatus* and *V. volvacea* increased in vitro DM digestibility at 24 h after incubation. Fermenting roughage with fungi did not affect rumen pH, which ranged from 6.60 to 6.91 (*p* > 0.05), while *P. ostreatus* resulted in increased levels of ruminal ammonia-nitrogen concentrations. Propionic acid increased in all roughages fermented with *P. ostreatus* or *V. volvacea* after 8 h of ruminal fermentation testing. The two fungi fermented as substrate treatments had significantly lower (*p* < 0.05) CH_4_ production. Based on the improved rumen DM digestibility and reduced CH_4_ production, *P. ostreatus* and *V. volvacea* could be utilized for enhancing feeding efficiency of roughage.

## 1. Introduction

In a tropical region, ruminants were fed mainly on agricultural crop residues or industrial byproducts [1]. Purple field corn residue is a new variety [2]. It contains a much higher amount of anthocyanin [3]. However, the use of corn residue as feed in animal production is limited due to its structure, low nutrient value, and high structural carbohydrate content [4]. Recently, many studies have been conducted on the various physical and chemical treatments to improve rice straw as ruminant feed. In addition, numerous methods of biological pretreatments have demonstrated that fermentation with other diet ingredients or components improves the utilization of roughage as diet for ruminants [5].

Basically, the key to improve the use of crop waste for ruminants is to overcome the inherent barriers to rumen microbe fermentation. White-rot fungi have been applied for the pretreatment of fiber [6]. This can effectively increase biomass digestibility and improve the nutritive value of crop residues. Therefore, white-rot fungi agents with high fiber digestion efficiency, low price, and environmentally friendly properties need to be explored [7].

*Pleurotus ostreatus* and *Volvariella volvacea* are the most popular cultivated edible mushrooms in Thailand. They can grow on rice straw and degrade structural carbohydrates in straw [8]. The potential of these fungi to reduce lignin indigestible cell wall contents and increase the digestibility of straw has been reported [8]. Lignin degradation involves the activity of enzymes created by fungi. These enzymes, called lignases, include lignin peroxidases, manganese peroxidases, and laccase [4,8]. Moreover, they can produce lovastatin, which is able to decrease the methanogenic population in rumen. Lovastatin is a natural compound produced by *P. ostreatus* and *V. volvacea*. It is able to inhibit HMG-CoA reductase, which is an enzyme that mainly interacts in isoprenoid synthesis and is essential for cell membrane formation in methanogenic archaea [8]. Furthermore, the HMG-CoA reductase inhibitors are able to specifically inhibit ruminal archaea bacteria, which results in reduced methane (CH_4_) production in the rumen [9].

Thus, it was hypothesized that *P. ostreatus* and *V. volvacea* fermenting with rice straw, purple field corn and cobs would improve nutrient utilization via enhanced digestibility and lower CH_4_ production due to the effects of the lovastatin compound. Thus, the aim of the current experiment was to investigate the effect of inoculation of two fungi belonging to the white-rot fungus type on feed value and ruminal fermentation characteristics.

## 2. Materials and Methods

This study was carried out at Tropical Feed Resources Research and Development Center, Khon Kaen University. The experiment was guaranteed by the Animal Ethics Committee of Khon Kaen University (permission No. ACUC-KKU 99/2560 and issued on January 17, 2019), based on the Ethics of Animal Experimentation of National Research Council of Thailand.

### 2.1. Preparation of Rice Straw, Purple Field Corn Residue, and Fungal-treated Substrates

Rice straw was harvested from local rice fields in the area of Khon Kaen, Thailand. Purple field corn residue (stover and cob) was collected from the Plant Breeding Research Center for Sustainable Agriculture, Faculty of Agriculture, Khon Kaen University, Thailand. Roughage sources were collected, cut into 2 to 3 cm lengths, and sun dried for 3 to 5 days. The sun-dried substrates were then collected in plastic boxes and preserved at 28 to 32 °C until used.

*P. ostreatus* and *V. volvacea* were obtained as commercial products and were used to inoculate the substrates. They were controlled on potato dextrose agar plates at 25 °C for 7 days, kept at 4 °C, and subcultured every two weeks. Spore suspension was prepared in 0.1% Tween-80 solution in approximately 10^7^ spores/mL concentration [4].

The arrangement steps for the ferment were concisely [8] as follows. First, dried rice straw and purple field corn residues were stored. Then, the substrates were mixed with about 200 g of roughage sources, and 200 mL of distilled water (consisting of 1% molasses and urea) was added to provide the moisture condition of approximately 50% dry matter (DM).

Second, the cultured boxes were moved to a closed room for 21 days and fermented under the condition of average temperature at 30 °C, which was considered a suitable condition for fungal growth on the rice straw and purple field corn residues. On the last day of fermentation, all bags were transferred from the fermentation room, and the substrates were sun dried for 3 to 5 days. The sun-dried spent substrates were stored in plastic boxes and kept in the laboratory room until used, following the method of Chanjula et al. [4].

### 2.2. Experimental Design

The experiment was carried out according to a completely randomized 3 × 3 factorial design: 3 roughage sources (rice straw, purple corn stover, and purple corn field cob) for 3 inoculation methods (untreated, *P. ostreatus* treated, and *V. volvacea* treated). For the control group, the untreated roughage sample fungi (not inoculated with fungi) were stored in the same manner with the other two fungi treatments. Gas production technique was used to evaluate rumen fermentation.

### 2.3. Chemical Composition Analysis

Twenty-one days after treatment, 200 g of fermented roughage was collected for analysis of DM, organic matter crude protein (CP) [10], neutral detergent fiber (NDF), and acid detergent fiber (ADF) [11]. Analyses of roughage chemical composition are reported in Table 1. In addition, the lovastatin was analyzed by the method used by Pattanagul et al. [12] and modified by Khampasan et al. [2]. A diet characterized by a roughage/concentrate ratio of 70/30 was used as the substrate. Substrates were milled to a 1 mm screen and weighed to 0.2 g of DM into 40 mL bottles for 96 h incubation to study the rumen fermentation using in vitro gas techniques. All treatments were run triplicated of blank in an incubation for three replications.

### 2.4. Ruminal Inocula

All processes between the ruminal fluid moving and inoculum periods were anaerobic techniques and sterile. Two male dairy steers with an initial body weight of 400 ± 20 kg were used as rumen fluid donors. Rumen fluid samples were collected from animals before feeding in the morning. The rumen fluid was filtered through four layers of cheesecloth into prewarmed thermos flasks. The fluid was moved to the laboratory.

### 2.5. Preparation of Medium Solution

Artificial saliva was prepared as follows according to the method of Menke and Steingass [13]. Artificial saliva and rumen fluid, respectively, were prepared and mixed in a 2:1 ratio to prepare the fermentation solution. All bottles with the mixture of substrate treatments were prepared in a water bath at 39 °C for 1 h before filling with 30 mL of the rumen inoculum mixture. During the incubation, the gas production was noted at 0, 1, 2, 4, 6, 8, 10, 12, 18, 24, 48, 72 and 96 h.

### 2.6. Fermentation Characteristics Determination

The rumen solution mixture was collected at 0, 4, 8, 12 and 24 h of incubating after inoculation. Rumen fluid inocula were then filtered through four layers of cheesecloth. Samples were separated into 3 portions; the first portion was used for ammonia-nitrogen (NH_3_-N) determination using the micro-Kjeldahl methods [10] and volatile fatty acid (VFA) analysis using HPLC [14]. Calculation of CH_4_ production in rumen using VFA proportions according to Moss et al. [15] as follows: CH_4_ production = 0.45 (acetate) − 0.275 (propionate) + 0.40 (butyrate). In vitro digestibility was measured after dissolving at 12 and 24 h after inoculum, when the sample was filtered through pre-weighed Gooch crucibles (40 mm of porosity) and DM residue was determined. The percentage of weight loss was determined and reported as in vitro DM digestibility (IVDMD) [16].

### 2.7. Measurement of Monomeric Anthocyanin Content (MAC)

Total monomeric anthocyanin content (MAC) in all samples, divided into stover and cob, was measured using the method of Lee et al. [17]. Concentration of MAC analysis using a UV-Vis spectrophotometer (GENESYS 10S, Thermo Scientific, Waltham, MA, USA). Total MAC, total MAC per stover dry weight of one ear (MAC/e), and total MAC per cob DM in one ear (MAC/e) were reported as milligrams of cyanidin-3-glucoside equivalents per 100 g dry weight (mg CGE/100 g DW) of samples.

### 2.8. Statistical Analysis and Equation

Cumulative gas production data were fitted to the model of Orskov and McDonald [18], and anthocyanin content was calculated by using the following equation of Lee et al. 2005 [17].

All data were used for the statistical analyses composed of 3 kinds of roughages, 2 kinds of white-rot fungi and untreated 3 replications and runs, making a total of 27 observations. All obtained data were conducted using PROC GLM of SAS version 9.0 (SAS Inst. Inc., Cary, NC, USA) [19] according to a 3×3 factorial arrangement in CRD. The statistical model including roughages sources, white-rot fungi type and interaction effects were: Yij = μ + Ai + Bj + ABij + εij; where Yijk is an observation, μ is the overall mean, A is roughages sources effect (*i* = 1, 2, 3), B is white-rot fungi type effect (*j* = 1, 2, 3), AB is the interaction effect of roughages sources and white-rot fungi type, and εij the residual effect. Multiple comparisons among treatment means were performed by Duncan’s New Multiple Range Test (DMRT) and orthogonal contrast [20].

## 3. Results and Discussions

### 3.1. Chemical Composition of Roughage

The chemical composition of rice straw with purple field corn residue (stover and cob) fermented with *P. ostreatus* and *V. volvacea* is shown in Table 1. The rice straw, purple field corn stover, and purple field corn cob untreated with fungi consisted of 3.0%, 4.9% and 3.3% CP, respectively. However, the CP content of the rice straw, purple field corn stover, and cob fermented with *P. ostreatus* or *V. volvacea* increased when compared with untreated rice straw with fungi. This was probably associated with the mycelia growth of fungi, which increased the concentration of protein [21]. Chanjula et al. [4], who reported that CP concentration of fungi-fermented oil palm fronds and fungi fermented oil palm fronds with 1% urea, had a similar finding of an increase after treatment with *Lentinus sajor-caju*. Furthermore, the CP content of the straw was developed by fermenting with *Pleurotus* species. In addition, there were similar CP contents when roughage was fermented with *P. ostreatus* or *V. volvacea* (*p* > 0.05).

Rice straw, purple field corn stover, and purple field corn cob fermented without fungi contained 79.8%, 80.6% and 86.7% NDF and 52.4%, 53.2%, and 45.7% ADF, respectively. However, the NDF and ADF that consisted of the rice straw, purple field corn stover, and purple field corn cob fermented with *P. ostreatus* and *V. volvacea* were reduced when compared with the untreated group. This could be an indication of cell wall breakdown by *P. ostreatus* and *V. volvacea*. The two fungi that are able to digest lignin by secondary metabolites such as lignin peroxidase, manganese peroxidase, and laccase enzyme [4]. The results showed that *P. ostreatus* or *V. volvacea* treatment increased the nutrient value of the straw by lowering its cell wall consistency as determined by its cellulose, hemicellulose, and lignin. The results agree with those of Rahman et al. [22] and Chanjula et al. [4], who found that cellulose, hemicellulose, and lignin were decreased in fungal-treated oil palm fronds and fungal-treated oil palm fronds with 1% urea when compared with a nonfermented group.

The lovastatin concentration in roughage treated with *P. ostreatus* and *V. volvacea* is shown in Table 2. There were interactions between sources of roughage and two fungi (*p* < 0.01). Type of fungi could affect the concentration of lovastatin when compared to the untreated group. In addition, *P. ostreatus* had higher potential to produce lovastatin than *V. volvacea* (*p* < 0.05). The maximum yield of lovastatin was obtained from rice straw fermented with *P. ostreatus*, which could be because the cellulose, hemicellulose and lignin in rice straw are weakly linked structures when compared with purple field corn by products. Therefore, *P. ostreatus*, which is a microbial agent with high lignocellulose degradation efficiency, can alter the physical structure. These reasons agree with those advanced by Mustafa et al. [23], who reported that these processes can improve the chemical content and structure of lignocellulosic materials, damaging the bond between polysaccharides and lignin, and thereby producing cellulose and hemicelluloses that are more accessible to hydrolytic enzymes [4].

The MAC in rice straw, purple field corn stover and purple field corn cob fermented with *P. ostreatus* or *V. volvacea* is reported in Table 2. The purple field corn variety had the highest MAC in cobs, positively and significantly ranging from −0.28 to −0.69 when compared with normal field corn [2]. This was similar to the finding of Khampasan et al. [2], who revealed that the MAC in purple field corn had higher genotypes (2022.1 mg CGE/100 g DW) than normal varieties. The MAC in untreated purple field corn cobs was higher than in the fermentation groups, which could be due to temperature and the fermentation process reducing the MAC content. Moreover, thermal stability of anthocyanins varied according to conditions such as pH and temperature [24]. Nevertheless, the pH status did not explain the influence of the MAC stability in the fermented roughage not continuing to decline. To realize anthocyanin stability during roughage-treated storage, the relationship between anthocyanin stability and lactic fermentation should be explained, because anthocyanin contains anthocyanidin and sugars, and there is a possibility that sugars, from anthocyanin, are used as a substrate for lactic fermentation. According to previous reviews [2], many factors affect the stability of anthocyanin. Among those factors, high pH, oxygen, high temperature, and light participate in the stability of the anthocyanin in the roughage treated during storage. However, the current study also showed that the anthocyanin in purple field corn was not decomposed by ruminal fluid, which shows that anthocyanin-rich corn is suitable to use as feed for ruminants [25]. Moreover, this result is essential for reducing corn production residues.

### 3.2. Production of Gas and Kinetic Gas Analysis

Cumulative gas production for all treatments was shown as gas production, and values for the determined parameters obtained from the kinetics of gas production models for the substrates studied are shown in Table 3. It was found that gas production from soluble fractions was different among treatments (*p* < 0.01). There was no interaction effect between roughage and *P. ostreatus* and *V. volvacea* on the insoluble fraction and the gas production rate constant for the insoluble fraction (*p* > 0.05). Regarding all treatments, the gas production rate was constant for the insoluble fraction and was significantly highest (*p* < 0.01) in the treatment in which purple field corn stover was fermented with fungi. This increased performance of kinetic gas could be determined to result in the highest CP of approximately 10.9% in stover, resulting in effective ruminal fermentation [26]. In contrast, *P. ostreatus* and *V. volvacea* treated roughage sources had interaction effects on the insoluble fraction and total gas production (96 h). Kinetic gas production was higher in the current study, which could be due to silage fermentation, improved rumen fermentation and nutrient digestibility [27,28]. White-rot fungi might disintegrate structure linkages of lignin, hemicellulose, and cellulose and thus, resulting enhance gas production [8].

### 3.3. In Vitro Digestibility

The effect of roughage fermented with *P. ostreatus* or *V. volvacea* on in vitro digestibility at 12 and 24 h is reported in Table 4. There was significant interaction in in vitro DM digestibility at 12 h of incubation. Purple field corn cob had a higher significant effect on in vitro DM digestibility at 12 and 24 h after incubation when compared to that of other groups. This could be due to less composed structural carbohydrates at 16.47% ADF in cobs when compared with rice straw and purple field corn stover. Similar to a report by Wachirapakorn et al. [29], who demonstrated that the hemicellulose structure content in ground corn cob was increased in rice straw at 13.02%, the cellulose contained in rice straw was lower at 4.5% DM and lignin was higher at 10% DM. Cellulose is generally known for its low palatability, poor digestibility, and insufficient nutrient availability [11]. In particular, the high ratio of lignin consent leads to slow rates of feed digestion. Moreover, current research has found that roughage fermented with *P. ostreatus* and *V. volvacea* increased in vitro DM digestibility at 24 h after incubation. The results from the in vitro gas technique have been used as an estimation of nutrient digestion, high gas production rate, and high digestibility of substrates [30]. In the current study, it was demonstrated that treatments, in which a high gas production rate appeared, were also found to result in higher in in vitro digestibility of DM. These results suggest that cobs treated with *P. ostreatus* had significantly greater lignin loss ratio.

### 3.4. Ruminal pH and Ammonia-Nitrogen (NH_3_-N)

The effect of fermenting roughage with *P. ostreatus* and *V. volvacea* on rumen pH and concentrations of ruminal ammonia-nitrogen (NH_3_-N) is reported in Table 4. Fermenting roughage with fungi did not affect rumen pH, which ranged from 6.60 to 6.91 (*p* > 0.05). The ruminal pH condition for all treatments was optimal for normal rumen fermentation, rumen microbe growth and their activity to digest fiber and feed [31]. On the other hand, *P. ostreatus* resulted in increased levels of NH_3_-N concentrations (approximately 60.29%), based on the fact that NH_3_ is the key end product of protein digestion in the rumen, and the fact, which has been shown to be generally agreed upon, that most of the N utilized by ruminal microbes comes from the NH_3_ pool in rumen [32,33,34]. In addition, the great content of NH_3_-N in the substrate treated with white-rot fungi was maybe the high CP content, which is an additional N source. In addition, these white-rot fungi contain protein and there is a probability that the protein of anthocyanin is used as a substrate for synthesis.

### 3.5. VFA and CH_4_ Production

The total VFA, acetic acid, propionic acid, and butyric acid proportions are shown in Table 5. The total VFA concentrations (4 hours after incubation) ranged from 93.64 to 102.05 mmol/L and were similar to concentrations represented by Wanapat et al. [35]. The concentrations of acetate, propionate, and butyrate in this study were in accordance with those reported by Wanapat et al. [35] and Wachirapakorn et al. [29]. They had an interaction effect on butyric acid and total VFA 4 and 8 h of incubation. Propionic acid was increased in roughages fermented with *P. ostreatus* and *V. volvacea* after 8 h of incubation. Moreover, propionic acid increased in treated purple field corn stover and cob after 4 h of incubation. This could be because *P. ostreatus* improved fiber digestibility and enriched anthocyanin in corn. This is contrary to the results of Hosoda et al. [3], who found that acetate, propionate and butyrate concentrations in swamp buffaloes were affected by anthocyanin-rich corn. The effect that anthocyanin presents in purple field corn is one of the phenolic compound, which could inhibit CH_4_ production by shifting hydrogen from the CH_4_ pathway to form propionic acid [26,33,36]. Gallic acid in corn might also lead to rechanneling of the hydrogen metabolism from CH_4_ to produce propionic acid. CH_4_ production in rumen was calculated using VFA according to Moss et al. [16], showed that *P. ostreatus* and *V. volvace*a fermented as substrate treatments had significantly lower (*p* < 0.05) CH_4_ production (8 hours after incubation), approximately 10.83% to 11.32% that of the untreated substrates (Table 6). However, there were no differences between *P. ostreatus* and *V. volvacea*. This was probably due to the effects of anthocyanin and gallic acid on propionic acid production. Moreover, lovastatin might have the similar negative affect the HMG-CoA reductase enzyme in white-rot fungi as it has in methanogen bacteria. Archaea produces CH_4_ in the methanogenesis processes, because generic fungi have developed to permit producer strains to obtain a competitive survival advantage by interfering with the assembly of isoprenoid chains required for membrane phospholipid synthesis [32,34,37]. Moreover, lovastatin might inhibit the expression of the F420-dependent NADH reductase gene in *Methanobrevibacter smithii*. The gene generates the enzyme for the move of the methyl group from methyl-H4MPT to HS-COM [36]. Methyl coenzyme-M reductase (mcr) is the key enzyme in the methanogenesis pathway. These results were supported by those of Jahromi et al. [8], who demonstrated that fermented rice straw containing lovastatin has the potential to reducing CH_4_ emission by 96.43%.

## 4. Conclusions

In conclusion, purple field corn stover fermented with *P. ostreatus* or *V. volvacea* could be utilized to enhance the feeding efficiency of roughage. However, further long-term studies on the feed and the economics of its use should be conducted using fermented *P. ostreatus* and *V. volvacea* as substrates in feeding trials, emphasizing lactating dairy cows and fattening beef cattle.

## Figures and Tables

**Table 1:** Chemical composition in rice straw, purple field corn stover, and cob.

Indices		DM (%)	OM (%DM)	NDF (%DM)	ADF (%DM)	CP (%DM)
Rice straw	Untreated	94.7	91.4	79.8	52.4	3.0
	*P. osteratus*	51.0	85.2	77.3	50.6	4.5
	*V. volvacea*	52.6	86.1	76.4	51.4	4.3
Purple corn stover	Untreated	94.4	96.5	80.6	53.2	4.9
	*P. osteratus*	54.5	90.6	78.6	51.4	5.6
	*V. volvacea*	53.6	91.3	78.4	51.6	5.5
Purple corn cob	Untreated	90.4	96.8	86.7	45.7	3.3
	*P. osteratus*	56.7	60.3	82.6	41.2	4.0
	*V. volvacea*	56.9	61.2	81.4	40.9	4.1
Contrasts						
Roughage		*	*	*	*	*
White-rot fungus type		*	*	**	**	*
Interaction		**	**	**	**	**

DM, dry matter; OM, organic matter; NDF, neutral detergent fiber; ADF, acid detergent fiber; CP, crude protein; SEM, standard error of the mean; **, mean indicating significant differences (*p* < 0.01); *, mean indicating significant differences (*p* < 0.05).

**Table 2:** Monomeric anthocyanin content (MAC) and lovastatin in rice straw, purple field corn stover and cob.

Indices		MAC (mg/100 g)	Lovastatin (mg%)
Rice straw	Untreated	0.60	0.00
	*P. osteratus*	0.55	39.00
	*V. volvacea*	0.45	31.55
Purple corn stover	Untreated	11.95	0.0
	*P. osteratus*	4.55	35.95
	*V. volvacea*	5.65	29.50
Purple corn cob	Untreated	139.40	0.00
	*P. osteratus*	34.25	32.00
	*V. volvacea*	39.50	30.95
Contrasts			
Roughage		*	*
White-rot fungus type		**	*
Interaction		**	**

SEM, standard error of the mean; **, mean indicating significant differences (*p* < 0.01); *, mean indicating significant differences (*p* < 0.05).

**Table 3:** Effect of roughage fermentation with white-rot fungi on gas kinetic kinetics and cumulative gas production after 96 h of incubation.

Indices		Kinetic of Gas, mL/ 0.5 g DM			Cumulative Gas Production, mL Gas/ g DM Incubated
		a	b	c	
Rice straw	Untreated	1.14	79.12	0.044	81.78
	*P. osteratus*	−0.67	75.11	0.051	71.43
	*V. volvacea*	−0.74	72.84	0.050	52.93
Purple corn stover	Untreated	−1.11	65.57	0.052	64.89
	*P. osteratus*	−2.47	66.62	0.064	62.73
	*V. volvacea*	−0.86	65.58	0.064	49.84
Purple corn cob	Untreated	−0.34	70.81	0.048	69.91
	*P. osteratus*	−0.58	71.60	0.050	70.06
	*V. volvacea*	0.59	71.80	0.053	71.17
Contrasts					
Roughage		*	*	*	*
White-rot fungus type		*	**	*	*
Interaction		**	ns	ns	**

a, the gas production from the immediately soluble fraction; b, the gas production from the insoluble fraction; c, the gas production rate constant for the insoluble fraction (b); SEM, standard error of the mean; **, mean indicating significant differences (*p* < 0.01); *, mean indicating significant differences (*p* < 0.05); ns, mean non-significant among treatment.

**Table 4:** Effect of roughage fermentation with white-rot fungus type on rumen ecology and in vitro digestibility.

Indices		NH_3_-N (mg/dl)	pH4	pH8	IVDMD12 (%DM)	IVDMD24 (%DM)
Rice straw	Untreated	3.60	6.87	6.83	56.15	58.22
	*P. osteratus*	12.26	6.81	6.77	58.02	67.66
	*V. volvacea*	11.90	6.83	6.76	58.73	68.50
Purple corn stover	Untreated	5.63	6.83	6.66	49.84	56.29
	*P. osteratus*	12.67	6.85	6.72	54.62	65.10
	*V. volvacea*	11.84	6.89	6.71	57.88	65.39
Purple corn cob	Untreated	5.71	6.85	6.71	60.08	61.96
	*P. osteratus*	12.69	6.91	6.60	67.69	69.53
	*V. volvacea*	11.78	6.90	6.66	63.31	67.88
Contrasts						
Roughage		*	*	*	*	*
White-rot fungus type		**	*	**	*	*
Interaction		ns	ns	ns	**	ns

NH_3_-N, ammonia-nitrogen; pH4, pH at 4 hours after incubation, pH8, pH at 8 h after incubation IVDMD12, in vitro dry matter digestibility at 12 h after incubation; IVDMD24, in vitro dry matter digestibility at 24 h after incubation; SEM, standard error of the mean; **, mean indicating significant differences (*p* < 0.01); *, mean indicating significant differences (*p* < 0.05); ns, mean non-significant among treatment.

**Table 5:** Effect of roughage fermentation with white-rot fungus type on total volatile fatty acids (TVFA) and VFA profiles.

		4 Hours after Incubation			Total VFA (mmol/l)	8 Hours after Incubation			Total VFA (mmol/l)
Indices (mmol/l)		C2 (%)	C3 (%)	C4 (%)		C2 (%)	C3 (%)	C4 (%)	
Rice straw	Untreated	73.42	17.95	11.52	102.05	71.88	18.57	10.25	100.02
	*P. osteratus*	68.53	19.85	9.69	99.26	68.97	21.15	8.55	98.77
	*V. volvacea*	67.84	20.16	9.78	98.59	68.08	21.11	9.51	99.12
Purple corn stover	Untreated	74.94	18.36	17.20	100.63	74.00	16.46	10.91	102.55
	*P. osteratus*	67.03	19.95	13.43	101.32	70.60	21.30	10.40	100.70
	*V. volvacea*	66.05	19.48	11.22	101.30	71.27	22.64	10.15	101.15
Purple corn cob	Untreated	60.41	20.53	7.71	96.86	60.85	19.41	9.77	96.27
	*P. osteratus*	59.66	22.05	6.78	93.64	59.17	21.82	8.09	95.11
	*V. volvacea*	58.05	20.99	6.25	95.10	58.68	21.98	8.19	95.01
Contrasts									
Roughage		*	*	**	*	*	*	*	*
White-rot fungus type		**	*	*	*	*	*	**	*
Interaction		ns	ns	**	ns	ns	*	ns	ns

C2, acetic acid; C3, propionic acid; C4, butyric acid; SEM, standard error of the mean; **, mean indicating significant differences (*p* < 0.01); *, mean indicating significant differences (*p* < 0.05); ns, mean non-significant among treatment.

**Table 6:** Effect of roughage fermentation with white-rot fungus type on methane (CH_4_) production.

Indices		Methane (CH_4_) Production (mL/L)	
		4 Hours after Incubation	8 Hours after Incubation
Rice straw	Untreated	32.08	32.03
	*P. osteratus*	29.92	28.54
	*V. volvacea*	29.50	28.54
Purple corn stover	Untreated	33.78	31.88
	*P. osteratus*	31.50	27.23
	*V. volvacea*	32.10	27.40
Purple corn cob	Untreated	29.04	27.52
	*P. osteratus*	27.62	26.37
	*V. volvacea*	27.71	26.55
Contrasts			
Roughage		*	*
White-rot fungus type		**	*
Interaction		ns	ns

SEM, standard error of the mean; **, mean indicating significant differences (*p* < 0.01); *, mean indicating significant differences (*p* < 0.05); ns, mean non-significant among treatment.
